# A Lossless Sink Based on Complex Frequency Excitations

**DOI:** 10.1002/advs.202301811

**Published:** 2023-08-16

**Authors:** Curtis Rasmussen, Matheus I. N. Rosa, Jacob Lewton, Massimo Ruzzene

**Affiliations:** ^1^ P. M. Rady Department of Mechanical Engineering University of Colorado Boulder Boulder CO 80309 USA

**Keywords:** coherent virtual absorption, complex frequencies, diffraction limit, wave sink, wave trapping

## Abstract

The creation of a sink in a lossless wave‐bearing medium is achieved using complex frequency signals—harmonic excitations that exponentially grow in time. The wave sink, where incident waves are confined to a point, has attracted interest for imaging and sensing since it may lead to arbitrarily small hotspots that surpass the diffraction limit. However, most methods of creating sinks require careful tuning, such as by impedance matching the sink to free space through the inclusion of loss, which imposes constraints on emerging applications. An alternative method, proposed here, relies on complex frequency excitations, bypassing the need to modify the scattering system by instead shaping the input signal. Eigenvalue zeros derived from a scattering formalism extended to the complex frequency plane reveal operating conditions that induce complete energy trapping under steady‐state conditions in a framework generally applicable to 2D and 3D media. To support the developed theory, an experiment is performed where a sink is realized using elastic waves on a plate with a circular cutout. These findings may lead to imaging and sensing applications relying on subwavelength focal points and nonlinear wave generation due to the high amplitudes achieved over short timescales.

## Graphical abstract

By considering waves with imaginary parts added to their frequencies, a lossless sink where incident wave energy is perfectly stored in a subwavelength inhomogeneity is introduced. The report provides a general recipe for the creation of such a sink, equally applicable to electromagnetics, acoustics, and elastodynamics. An experiment with vibrations in an aluminum plate demonstrates the findings.

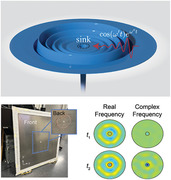

## 1 Introduction

The point source, the mathematical abstraction of an infinitesimally small wave generator, enjoys a long history in the analysis of electromagnetic and acoustic systems. In linear systems, the point source's analytical power comes from the superposition principle, which states that the total response from an extended source is simply the addition of the fields emitted from an array of point sources. Less commonly considered is the sink, where incident wave energy is fully absorbed at a point. Although the creation of wave sinks is not as straightforward as the creation of a source, they are intriguing for their ability to create subwavelength hotspots. This has implications for the resolution limit in imaging systems. For instance, the Abbe diffraction limit imposes a strict bound on the width of a focal spot tied to the wavelength.^[^ ^1^, ^2^ ^]^ Attempts at overcoming this limit include converting scattered evanescent fields into propagating ones^[^ ^3^, ^4^, ^5^ ^]^ and super‐oscillation techniques,^[^ ^6^ ^]^ both of which typically suffer from high losses, and are limited to near‐field sensing,^[^ ^7^ ^]^ where the sensing probe is required to be very close to the object being imaged.

In this context, it has been recently recognized that the presence of a wave sink may lead to subwavelength focusing^[^ ^8^, ^9^ ^]^ and surpass the diffraction limit, allowing for far‐field imaging that avoids high loss. An example is the case of Maxwell's fisheye lens, where perfect resolution is possible with a point wave sink.^[^ ^10^ ^]^ Currently, the generation of wave sinks requires the careful tuning of impedance matching parameters. For example, prior works have shown that a sink can be made from a scatterer whose impedance is perfectly matched to that of a converging wave.^[^ ^8^, ^9^, ^11^ ^]^ This approach requires that loss be introduced into the system. In the context of the scattering matrix formalism relating input and output energy channels, this can be understood as an example of coherent perfect absorption (CPA) with a single radiation channel.^[^ ^12^, ^13^, ^14^, ^15^, ^16^, ^17^, ^18^ ^]^ It has also been shown how a converging wave can be canceled out by using a source simultaneously emitting the inverse of the incident signal in much the same way that noise‐canceling headphones are able to quiet a noisy room.^[^ ^19^, ^20^, ^21^ ^]^ These approaches match the intuition that some amount of energy conversion is required to dispose of the impinging energy.

We here propose an alternative method where energy is instead perfectly stored within the sink without requiring tuned loss or indeed any tuning of the scattering system whatsoever. Recent work has shown how the use of complex frequency excitations, with input signals either increasing or decreasing in time, unlocks the ability to mimic non‐Hermitian effects associated with gain and loss, such as PT symmetry,^[^ ^22^ ^]^ critical coupling,^[^ ^23^ ^]^ optical pulling,^[^ ^24^ ^]^ and the non‐Hermitian skin effect.^[^ ^25^ ^]^ This technique is referred to as coherent virtual absorption (CVA) and is in contrast to CPA that employs real frequencies. CVA was first proposed in electromagnetics^[^ ^26^ ^]^ and was later demonstrated in a 1D acoustic system.^[^ ^27^ ^]^ It has also been analyzed for photonic micro ring resonators.^[^ ^28^, ^29^ ^]^ These various implementations effectively operate as if loss or gain are encoded in the excitation signal itself, which can be appealing for the realization of non‐Hermitian phenomena,^[^ ^30^, ^31^, ^32^, ^33^, ^34^, ^35^, ^36^ ^]^ where loss or gain elements are often bulky or difficult to realize. However, the CVA approach has not yet been applied to the case of the sink, which we pursue herein. The required frequencies are obtained through a scattering formalism extended to the complex frequency plane, where eigenvalue zeros show operating conditions with zero scattering from an inhomogeneity. By allowing no energy to leave, the wave sink acts as a storage cavity for the impinging waves. However, this storage cavity can be made vanishingly small as we show and may contain a hotspot with an amplitude that can in principle grow without bound. After first describing how any axisymmetric resonator can act as a sink at an infinite number of discrete complex frequencies, we demonstrate the realization of such a sink in an elastic plate, where mechanical waves converge on a symmetric cutout and are effectively trapped throughout the duration of the incoming signal.

## 2 Results

### 2.1 Concept

**Figure 1:**
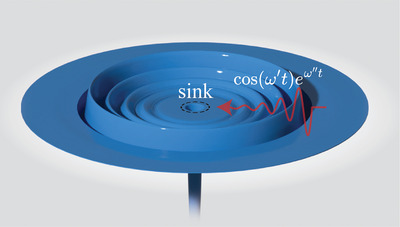
Complex frequency sink. Schematic showing a lossless resonator with the ability to perfectly confine waves of complex frequency ω. The real part of the frequency, ω′, dictates the oscillatory motion of the wave while the imaginary part, ω″, dictates the rate of exponential growth.

We consider a lossless circular inclusion with circularly symmetric waves incident on it as shown in **Figure** **1**. As the inclusion (dashed black line) is lossless, any real frequency excitation leads to unitary scattering, meaning that in steady‐state conditions the same amount of wave energy that is incident onto the inclusion is scattered away from the inclusion. This balance between input and output energy can be broken by the addition of loss in the inclusion and, in particular, if an amount of loss exactly related to the impedance of the traveling wave is introduced,^[^ ^5^ ^]^ the incident wave can be completely absorbed by the inclusion. This wave sink realized through the addition of loss is an example of CPA with one radiation channel.

We propose a novel sink based instead on CVA—that is keeping the inclusion lossless while using a complex frequency excitation. In order for it to act as a sink (i.e., no scattering) in the absence of loss, it needs to perfectly store the incident wave. Remarkably, there exists an infinite number of exponentially increasing sinusoidal signals that lead to this perfect‐storage condition. In Figure 1, we illustrate how a symmetrically converging wave of complex frequency ω = ω′ + *i*ω″ as given by Re[e−i(ω′+iω′′)t]=cos(ω′t)eω′′t may enter without reflection in the central sink region, leading to a wave amplitude in the sink that grows without bound as long as the input signal is maintained. The complex frequency excitation is composed of two parts. One, a harmonic oscillation governed by ω′, the real part of the complex frequency and, two, an exponential growth governed by ω″, the imaginary part. The sink operates at specific combinations of ω′ and ω″, as we will illustrate both numerically in the context of the Helmholtz equation and experimentally with elastic waves on a metal plate in the following sections.

While in the sketch of Figure 1, the size of the sink is on the order of the size of the wavelength, we will show that, within the context of waves governed by the Helmholtz equation, complex frequencies can be used to create an arbitrarily small subwavelength lossless sink. And we will discuss the scattering formalism for the phenomena, which provides a framework for finding the required complex frequencies, with applicability to a wide variety of wave physics, such as electromagnetic, acoustic, or elastodynamic, 2D or 3D.

### 2.2 Theory

**Figure 2:**
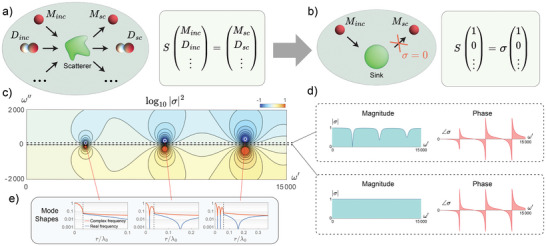
Theoretical framework of the wave sink. a) Scattering formalism where the scattering matrix *S* relates incident and scattered monopolar, dipolar, and higher‐order modes from a generic scatterer. b) The condition of zero eigenvalue σ leads to zero scattered energy from an incident monopole on an axisymmetric scatterer. c) Reflection coefficient landscape showing the first three zero‐reflection complex frequencies. The magnitude of σ is the ratio between the output and input signals. As shown in d) this magnitude takes a value of one along the real axis, with a phase that swings as the scattering zeros are crossed. The magnitude can, however, be less than one and even become zero at discreet frequencies in the upper half complex plane. e) The shape of the complex standing waves present in the sink, along with the fields outside the sink, shown for the first three zero‐reflection frequencies. The dashed lines indicate the outer edge of the sink.

The operation of the complex frequency sink is understood through the scattering matrix formulation of an inclusion in a homogeneous medium, relating incident wave modes to scattered wave modes.^[^ ^37^, ^38^ ^]^ Using a 2D cylindrical basis, the scattering matrix relates how much monopole, dipole, and higher‐order multipole waves are scattered by an inclusion due to any pattern of incident waves. This is illustrated in **Figure** **2**a for a generic scatterer of arbitrary shape, where incident (_inc_) and scattered (_sc_) monopoles (*M*), dipoles (*D*), and so on are represented in the *S* matrix formalism, by $S{\left(\begin{array}{ccc} M_{\text{inc}} & D_{\text{inc}} & \text{…} \end{array}\right)}^{T}={\left(\begin{array}{ccc} M_{\text{sc}} & D_{\text{sc}} & \text{…} \end{array}\right)}^{T}$. The scattering matrix *S* is defined by the geometry and material properties of the inclusion and describes how much of each multipole the inclusion scatters. Its dimensionality corresponds to the number of multipoles being considered.

To create a sink, we require that no energy is scattered from the inhomogeneity, which is expressed as $S{\left(\begin{array}{ccc} M_{\text{inc}} & D_{\text{inc}} & \text{…} \end{array}\right)}^{T}=0$. This can be achieved by considering the eigenvalue problem $S{\left(\begin{array}{ccc} M_{\text{inc}} & D_{\text{inc}} & \text{…} \end{array}\right)}^{T}=\sigma {\left(\begin{array}{ccc} M_{\text{inc}} & D_{\text{inc}} & \text{…} \end{array}\right)}^{T}$, and looking for frequencies which make the eigenvalue zero, that is, σ = 0. At such frequencies, zero scattering follows from a particular pattern of incoming waves given by the corresponding eigenvector. In CPA, a specific amount of loss is added to the system to make a zero eigenvalue land on a real frequency value.^[^ ^12^, ^13^ ^]^ We here keep the system lossless and instead engage the complex frequency values for which σ = 0, a newer technique often referred to as CVA.^[^ ^26^, ^27^ ^]^

For an inhomogeneity of arbitrary shape, the zero eigenvalue may require a complicated pattern of incoming waves associated with its eigenvector. To simplify the problem we consider an axisymmetric scatterer with isotropic material properties, which avoids any coupling between multipoles, as illustrated in Figure 2b. In this case the scattering matrix is diagonal and any incoming monopole is reflected as an outgoing monopole, incoming dipole as an outgoing dipole, and so on. Since the scattering matrix is now diagonal, ${\left(\begin{array}{ccc} 1 & 0 & \cdots \end{array}\right)}^{T}$ is an eigenvector of the scatterer. The eigenproblem is now simplified to the form $S{\left(\begin{array}{ccc} 1 & 0 & \cdots \end{array}\right)}^{T}=\sigma {\left(\begin{array}{ccc} 1 & 0 & \cdots \end{array}\right)}^{T}$, and the eigenvalue becomes a reflection coefficient, that is, σ = *M*_sc_/*M*_inc_. We can then examine its variation in the complex frequency plane to scan for the frequencies where it becomes zero.

We proceed by considering the general case of waves governed by the time‐harmonic Helmholtz equation

(1) $$\left(\nabla^{2}+k^{2}\right)\psi \left(r,\omega \right)=0$$

where *k* = ω/*c* is a non‐dispersive wavenumber, with ω denoting the angular frequency and *c* the phase velocity, while ψ(**r**, ω) is the field of interest as a function of position **r** and frequency. We consider this formulation as it finds wide application in wave propagation phenomena including acoustics, electromagnetic, and elastic waves. We consider a 2D geometry with a circular inclusion of radius *a* centered at the origin, whose properties are different from those of the surrounding medium and examine scattering from an axisymmetric input, where only the monopole (circularly symmetric) component is present. In this axisymmetric problem, the fields outside (ψ_0_) and within (ψ_1_) the inhomogeneity are expressed as

(2) $$\begin{array}{cc} \psi_{0} & =M_{\text{inc}}H_{0}^{\left(2\right)}\left(k_{0}r\right)+M_{\text{sc}}H_{0}^{\left(1\right)}\left(k_{0}r\right) \end{array}$$

(3) $$\begin{array}{cc} \psi_{1} & =A_{\text{sink}}J_{0}\left(k_{1}r\right) \end{array}$$

In the expressions above, *r* is the radial coordinate, *k*_*i*_ = ω/*c*_*i*_ is the wavenumber in medium *i*, $H_{0}^{\left(j\right)}$ is the zero‐order Hankel function of the *j*th kind, and *J*_0_ is the zero‐order Bessel function.

The field outside the axisymmetric sink is composed of an incoming monopole wave of amplitude *M*_inc_ and a scattered monopole wave of amplitude *M*_sc_. We then write the outgoing wave amplitude in terms of the desired eigenvalue σ according to *M*_sc_ = σ*M*_inc_. While the process can be carried out for any wave physics described by the Helmholtz equation, we specialize now to an example from acoustics for purposes of illustration, with ψ_0_ and ψ_1_ as acoustic pressure fields. By enforcing continuity of pressure (ψ_0_ = ψ_1_) and particle velocity (∂_*r*_ψ_0_/ρ_0_ = ∂_*r*_ψ_1_/ρ_1_) at the inhomogeneity boundary *r* = *a*, we arrive at this expression for the scattering eigenvalue σ

(4) $$\sigma =\frac{M_{\text{sc}}}{M_{\text{inc}}}=\frac{\rho_{1}k_{0}J_{0}\left(k_{1}a\right)H_{1}^{\left(2\right)}\left(k_{0}a\right)-\rho_{0}k_{1}J_{1}\left(k_{1}a\right)H_{0}^{\left(2\right)}\left(k_{0}a\right)}{-\rho_{1}k_{0}J_{0}\left(k_{1}a\right)H_{1}^{\left(1\right)}\left(k_{0}a\right)+\rho_{0}k_{1}J_{1}\left(k_{1}a\right)H_{0}^{\left(1\right)}\left(k_{0}a\right)}$$

This equation predicts how much the acoustic wave will be scattered by the sink at any frequency, real or complex, depending on the size of the sink and the material properties of the sink and the surrounding medium.

Considering the ambient medium to be air (sound speed *c*_0_ = 343 m s^−1^ and density ρ_0_ = 1.21 kg m^−3^), and considering a resonator of size *a* = 1 cm with properties *c*_1_ = 14.8 m s^−1^ and ρ_1_ = 74.6 kg m^−3^, we use Equation (4) to examine the amount of scattering from the resonator at any frequency in the complex frequency plane. This is done by plotting log_10_|σ|^2^ as a function of the real (ω′) and imaginary (ω″) parts of the frequency as shown in Figure 2c. In the bottom half of the complex frequency plane, scattering poles for which σ → ∞ are observed (red‐colored spots) at complex frequencies that make the denominator of Equation (4) equal zero. These frequencies lead to maximization of scattering from the resonator, a condition known as lasing when it occurs on the real frequency axis (see, e.g., ref. [37]). The upper half plane contains zeros (σ = 0) at complex frequencies which make the numerator of Equation (4) equal zero, which come in complex conjugate pairs to the poles as the system has no loss or gain. At these operating frequencies zero scattering from the resonator is achieved, which is the sought‐after CVA condition. As shown in Figure 2d, the magnitude of σ along the real frequency axis is always one due to conservation of energy in our conservative system: all energy incident on the lossless resonator is necessarily scattered by the resonator in steady‐state conditions. However, while the eigenvalue is always one, the phase of σ is free to take on any value and, in particular, it passes through resonant phase shifts along the real axis at points below the zero‐reflection complex frequencies. This observation is later exploited to find the zero‐scattering frequencies experimentally.

In general, an infinite number of such poles and zeros are expected from any set of generic properties of the medium and of the inclusion, with Figure 2c displaying the first three pairs for the selected example. Equating the numerator of Equation (4) to zero yields a transcendental equation that can be numerically solved to find the complex frequency zeros. In particular, the solution space of this equation can be explored to enforce specific operation conditions of the sink, as described in the Supporting Information. For the example in Figure 2c, we enforced the condition that the size of the sink be small relative to the wavelength of waves propagating at the complex frequency zero, that is *a*/λ_0_ < 1. We do this by setting Re[k0]a=0.1 in Equation (4), corresponding to *a*/λ_0_ = 0.1/2π.

We also enforce that the ratio between the real part of the frequency and the imaginary part of the frequency be ω′/ω″ = 40 at the first complex frequency zero. We did this for ease of demonstration: if the real part is overly large relative to the imaginary part the input signal looks very similar to a real frequency signal, but if the real part is overly small compared to the the imaginary part, the signal is dominated by the exponential increase and it is hard to visualize the wave dynamics. We found the value 40 to be a good balance for the following numerical demonstration. After setting these parameters, the sink size and its properties were determined as the remaining parameters were found to satisfy the transcendental equation. While we enforced these conditions to obtain a subwavelength sink, the procedure detailed in the Supporting Information can be generically applied to enforce other operating conditions, such as specific real or imaginary frequency values for the zeros or specific impedance ratios between the two media.

The subwavelength spot is formed by a radial standing wave in the complex frequency sink, mathematically described by a Bessel function with complex argument, which we plot on a semilog scale in Figure 2e. The plot shows the radial profiles of the standing wave at the first three zero‐reflection complex frequencies and corresponding real frequencies. For the real frequencies there are minima of zero amplitude within the resonator, while the minima remain nonzero for the complex frequencies. Each higher‐order zero‐reflection frequency has more minima, with the main lobe narrowing. These mode profiles clearly evidence the subwavelength nature of the sink, with the main radial lobe fully contained within the resonator. The spot size can be characterized by its full width at half maximum (FWHM), which is equal to 0.0078λ_0_ for all complex frequency zeros and their corresponding real frequencies.

While the spot size relative to a wavelength remains constant with order number, the size of the sink relative to a wavelength increases with order number, as seen for the three zeros plotted in Figure 2e, with the dashed lines indicating the outer edge of the sink. At high enough frequency, a subwavelength hotspot can be obtained even when the sink is no longer subwavelength. For example, at the zero‐reflection frequency 39,851 + 92.35i Hz, the 54th zero of the system, the ratio of the radius of the inhomogeneity to the wavelength is 1.16 while the FWHM of the spot relative to a wavelength is still 0.0078. Though, depending on the practical implementation of the sink, such a high‐order zero may be difficult to engage. The FWHM relative to wavelength is dictated by the material parameters of the system. In principle, the spot size can be made arbitrarily small if the material properties of the sink can be fully controlled. In practice, the limiting factors on the spot size are the ability of the sink to take on the required material parameters while also ensuring that the complex frequency required is feasible.

We note that, while both real and complex frequencies achieve the subwavelength condition, the fields outside the resonator are very different. The complex case exhibits only incoming radiation, which results in a uniform decay away from the origin characterized by the Hankel function $H_{0}^{\left(2\right)}$, while the real case has both incoming and outgoing radiation, which results in an oscillatory field that would be described by a Bessel *J*_0_ function. Hence, the complex frequency is capable of creating a subwavelength focal spot with zero scattering since it acts as a sink, while the real frequency will be necessarily accompanied by scattering which limits its ability to accumulate energy at the focal spot.

Physical intuition for the zero‐scattering process can be obtained by considering the destructive interference at play. At the zero‐scattering complex frequency, the portion of the wave leaving the sink is precisely equal and opposite in phase to the incident wave bouncing off the boundary at that moment. And, as the amplitude of the wave in the sink grows in time, the input complex frequency signal must grow in time, meaning that ω″ is necessarily greater than zero.^[^ ^23^ ^]^

**Figure 3:**
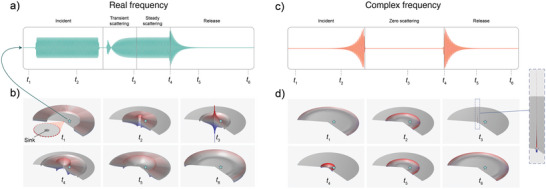
Subwavelength sink simulations. a) The time signal seen at a point (denoted by the blue star in the lower panels) in a wave‐bearing medium as a time‐limited real frequency wave is incident on a small sink region. Four distinct portions of the signal can be identified. 1) The incident signal as it approaches the sink. 2) The transient portion of the signal as some amount of the initial waves are scattered from the sink and, subsequently, as the wave energy in the resonator builds up. 3) The steady‐state portion where the resonator has reached the maximum amount of energy it can contain and all extra incident energy is balanced by an equal amount of outgoing wave energy. 4) With no more wave energy arriving at the sink, the energy built up in the resonator is released and an exponential decay is seen. b) Snapshots in time of the full wave field with the instantaneous wave amplitude represented by height and color. The times of the snapshots are indicated in (a). Boundary conditions on the outer rim are such that waves are free to enter and exit the domain with no extraneous reflections. c,d) Corresponding results for complex frequency excitation, where no scattering is seen in the signal. The energy contained by the sink grows without bound. When the incoming wave is turned off, the energy is released as the reverse signal to what was initially incident on the sink.

To validate the predicted zero scattering, we carry out full wave simulations of our system at the first complex frequency zero of our system (the left‐most blue dot in Figure 2c). The complex frequency monopolar wave is launched toward the axisymmetric resonator while the reflected signal is probed. We perform the simulation in the time domain, where the transient effects of the signal start and end can also be monitored. **Figure** **3** shows the results of the complex frequency cos (ω′*t*)exp (ω″*t*) simulation and a corresponding real frequency cos (ω′*t*) simulation for reference.

For the real frequency input (Figure 3a,b), different portions of the reflected waveform can be identified. The transient portion is characterized by an initial reflected signal followed by a buildup in amplitude showing that the energy is accumulating in the resonator. Next, the steady‐state portion of the signal where the resonator has reached its maximum amplitude and any further energy incident on the resonator is fully reflected. The reflected harmonic signal is no longer changing in time and the ratio of the amplitudes of the incoming and scattered signals is exactly one. This is the behavior seen for any real frequency input since, as observed in Figure 2d, |σ| = 1 along the entire real axis for our loss‐ and gain‐free system. The third portion of the waveform is the release of energy from the resonator which occurs after the signal is turned off. Figure 3b shows the full wave field at the instances in time indicated in the time trace of Figure 3a, which is the time history taken from the starred point.

Looking now at the complex frequency case corresponding to the predicted zero‐scattering condition, we see that only one portion of the reflected signal remains—the release of the energy from the resonator after the signal is turned off. While there is a small amount of transient scattering due to the cut on effects from the signal start, there is no steady‐state reflected portion of the signal. The snapshots in Figure 3d show the evolution of the simulation. In the first and second panels the incident wave is seen approaching the sink. The novel behavior, unachievable with real frequency excitations, is highlighted by the third panel, where the energy is completely contained within the lossless resonator before it is subsequently released back out as the incoming radiation is limited in length. To further visualize the evolution of the field in time, animations are included in the Supporting Information. These animations show the signal propagating into the resonator with no scattering. The wave amplitude in the resonator grows throughout the duration of the signal which creates a subwavelength focal point that increases without bound.

The resonator has been enforced to be subwavelength according to the real part of the incoming complex frequency signal, but it is still subwavelength when considering the highest component of the Fourier spectrum of the complex frequency waveform. The Fourier transform of a complex frequency signal from a time *t*_0_ to a time *t*_1_ is given by $F\left(\hat{\omega }\right)=\int_{t_{0}}^{t_{1}}\cos\left(\omega^{\prime }t\right)e^{\omega^{\prime \prime }t}e^{-i\hat{\omega }t}dt$. Under the assumptions that the input signal is of sufficiently long duration and that the real part of the complex frequency is large compared to the imaginary part, the power spectrum reduces to the simple Lorentzian form $|F\left(\hat{\omega }\right){|}^{2}=1/\left({\left(\hat{\omega }-\omega^{\prime }\right)}^{2}+\omega^{\prime \prime 2}\right)$. For the waveform used here, this expression is found to accurately describe the signal's spectrum and with this we can examine the degree to which the resonator is subwavelength at the highest frequencies present. We find that *a*/λ_0_ = 0.0163 at the half‐power (3 dB down) upper frequency and 0.0199 for the 20 dB down upper frequency.

While the analysis has concentrated on capturing incoming monopolar waves, we note that this is the condition that leads to the best performance a subwavelength sink can achieve for any general excitation, including that of a plane wave. This is because subwavelength scattering is dominated by the monopolar response, regardless of the shape of the sink. For example, in our subwavelength sink, the scattering from a plane wave at the first complex frequency zero is found (following the mathematical formulation described in ref. [39]) to contain over 60 times more scattered monopole than dipole with higher‐order multipoles being even more negligible. In this way, the best performance of a wave sink, a subwavelength particle to capture wave energy, in a plane wave field is achieved by tuning the particle to capture monopolar radiation, as considered here.

### 2.3 Experimental Implementation of a Complex Frequency Sink

**Figure 4:**
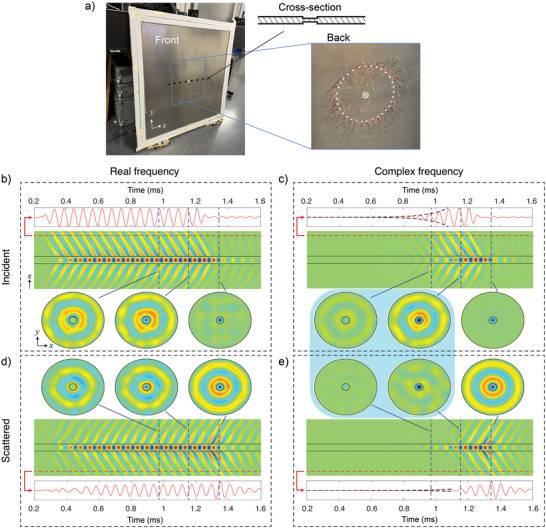
Experimental realization of a complex frequency sink for elastodynamic waves in a plate. a) An array of piezoelectric transducers launch a circular wave toward a pocket carved into an aluminum sheet. The vibration field is mapped using a laser Doppler vibrometer on the opposite side from the piezos in the area within the outer ring of piezos. b ,d) Measured results for real frequency excitation, where the excitation signal is the real part of the complex frequency signal used to obtain the results in panels (c) and (e). For insight into the function of the sink, the full vibration fields are decomposed into incident and scattered fields as described in the Experimental Section. The red time traces show the signals, incident and scattered, as measured at a point just within the ring of piezos. Waterfall plots show the wave amplitude as a function of time along a cross‐sectional cut of the full data. Snapshots of the full circular measured domain are also shown, with the sink area denoted by a black circle. For both real and complex frequency excitation, the third snapshots show the field after the incident signal is turned off. In these cases, there are no longer any incident waves and monopolar scattering is seen as the energy leaks out of the resonator. The first two snapshots show the difference between the real and complex frequency excitations. For the real frequency, these snapshots show an almost equal amount of wave energy exiting the sink as entering it, consistent with the scattering system being in the steady‐state condition. However, for the complex frequency excitation (highlighted with a blue background), very little scattered field is seen, while the incident field continues to grow exponentially in time. All plots are normalized such that the scattered and incident results are directly comparable.

The developed theory is widely applicable to any type of medium supporting linear waves. We experimentally verify these predictions by exploiting elastic waves propagating on a thin plate. To this end, we engrave a circular pocket in an aluminum plate as detailed in the Experimental Section. The setup is shown in **Figure** **4**a, where an axisymmetric input field is generated by a circular array of piezoelectric transducers. The full wave field is recorded with a scanning laser Doppler vibrometer, which captures the plate out‐of‐plane motion.

Differently from the Helmholtz wave equation, elastic waves in thin plates are governed by a fourth‐order differential equation, which according to Kirchhoff plate theory is expressed as^[^ ^40^ ^]^

(5) $$D\nabla^{4}w\left(r,t\right)+\rho h\frac{\partial^{2}w\left(r,t\right)}{\partial t^{2}}=0$$

where *w* is the bending displacement, ρ is the mass density, *h* is the plate thickness, and *D* = *Eh* ^3^/12(1 − ν^2^) is the bending stiffness, with ν representing the Poisson's ratio. We seek axisymmetric solutions to Equation (5) as we are considering the case of incident and scattered monopolar waves. We also want the solutions outside the resonator to be in the form of traveling waves while within the resonator we seek standing wave solutions. These considerations lead to ^[^ ^41^ ^]^

(6) $$w=\left\{\begin{array}{cc} M_{\text{inc}}H_{0}^{\left(2\right)}\left(k_{0}r\right)+M_{\text{sc}}H_{0}^{\left(1\right)}\left(k_{0}r\right)+A_{\text{evan},0}K_{0}\left(k_{0}r\right) & r>a\\ A_{\text{disk}}J_{0}\left(k_{1}r\right)+A_{\text{evan},1}I_{0}\left(k_{1}r\right) & r<a \end{array}\right.$$

where $H_{0}^{\left(2\right)}$ (the zero‐order Hankel function of the second kind) is the incoming wave, $H_{0}^{\left(1\right)}$ (the zero‐order Hankel function of the first kind) is the scattered wave, and *J*_0_ (the zero‐order Bessel function of the first kind) is the standing wave in the resonator. These terms are equivalent to the solution ansatz for the Helmholtz equation described in the previous section, with a different dispersion for Kirchhoff plates given as $k_{i}=\sqrt{\omega }{\left(\rho h_{i}/D_{i}\right)}^{1/4}$.^[^ ^41^ ^]^ In the case of the plate, additional evanescent solutions are present, which come from the fourth‐order derivative (in contrast to the second‐order derivative in the Helmholtz case). As such, *K*_0_ (the zero‐order modified Bessel function of the second kind) corresponds to an evanescent mode of amplitude *A*_evan, 0_ in the medium outside the resonator, while *I*_0_ (the zero‐order modified Bessel function of the first kind) is an evanescent mode with amplitude *A*_evan, 1_ present within the resonator.

Similarly to the acoustics case, by applying the appropriate boundary conditions at the resonator interface we can extract the reflection coefficient σ = *M*_sc_/*M*_inc_ between the non‐evanescent field components, and look for the existence of poles and zeros (see Note S4, Supporting Information). Indeed, numerical predictions of our thin plate setup evidence a zero‐reflection point at a complex frequency that is readily achieved in the lab. We run a test to determine more precisely its location in the complex frequency plane as any imperfections related to manufacturing or the simplified theory may shift its location. To this end, we exploit the fact that there is a phase swing in the scattered signal when the resonator is excited by a real frequency input (as shown in Figure 2d), recalling that while the amplitude of the reflected signal is always one, the real and imaginary parts vary as a function of real frequency. To achieve this goal, we employ real frequencies, extract the scattered signals, and fit the shape of the phase response to a simple complex zero at frequency ω_c_ with $\sigma =\left(\omega -\omega_{c}\right)/\left(\omega -\omega_{c}^{*}\right)$, as shown in the Supporting Information (see, e.g., ref. [16]). As a final step to ensure minimum reflection, we manually search the complex frequency space near this fitted frequency to arrive at the complex frequency with the least amount of scattering.

Videos of the full wave field corresponding to these results are presented in the Supporting Information. Representative time snapshots are shown in Figure 4 for both complex and corresponding real frequency inputs. As the measured field is a superposition of incoming and outgoing waves, frequency/wavenumber filtering procedures are employed to separate the waves propagating in opposite directions, as described in the Experimental Section, which allow us to accurately assess the amount of scattering. For real frequency excitation, the incoming and outgoing fields are approximately equivalent in amplitude, indicating that the attenuation in our metal plate is small and the system is adequately considered as lossless.

As highlighted by the blue box in panels (c) and (e), very little outgoing radiation is present for the complex frequency. This has been achieved without the introduction of carefully tuned loss elements, which have so far been the only viable approach to creating a wave sink. The time traces of the incoming and scattered signals at a point just outside the resonator, plotted in red, further confirm the effect. We see that while the waves are being launched into the resonator, only a small amount is reflected. In addition, the observed reflection, shown in the middle snapshot of Figure 4e, is mostly non‐monopolar (i.e., not circularly symmetric), caused by the (small) non‐monopolar components of the incident field that originate from slight differences in wave amplitude induced by the different piezoelectric discs as well as by any slight imperfections in the machining of the plate. As expected, when the signal is turned off, the waves are released from the resonator and are seen in the scattered signal. The scattered field after the excitation signal is turned off is almost perfectly monopolar (see last snapshot in Figure 4e and animation in Supporting Information), further confirming that the monopole component of the incident wave field is almost entirely trapped.

By fitting exponential envelopes to the signals we can obtain an estimate of the resonator's performance as a sink by taking the ratio of the amplitudes of the exponential signals. The amplitude of the incident wave, |*M*_inc_|, is estimated as shown in the time trace of Figure 4c by fitting the black dashed curve to the portion of the curve that is in steady‐state conditions. The curve is of the form |*M*_inc_|*e* ^2π1400*t*^ as the imaginary part of the frequency is 1400 Hz and the amplitude |*M*_inc_| that best fits the data is then found. The same procedure is used to estimate |*M*_sc_|, with the fit curve shown in Figure 4e. By dividing these values, we arrive at a calculation for the magnitude of the eigenvalue: |σ| = |*M*_sc_|/|*M*_inc_| = 0.11. We can use this value to estimate the amount of energy being captured in the resonator under the assumption that the majority of the incident and scattered wave energy is contained in the monopolar (circularly symmetric) mode. Visual inspection of the 2D field plots in Figure 4 show that this assumption makes for a fair approximation, under which the incident and scattered total wave energies are proportional to the absolute squares of the *M*_inc_ and *M*_sc_ values. From this, we can then estimate that approximately 99% of the incident energy (1 − |σ|^2^) is being captured in the resonator.

## 3 Conclusions

Our work reveals how waves can be trapped in a lossless sink through complex frequency excitation. We outline the general requirements and techniques for creating a wave‐trapping sink, drawing attention to how such sinks can even be made deeply subwavelength, and validate the proposed formulation in a table‐top demonstration employing elastic waves in a plate.

Notably, the use of complex frequencies for wave trapping can allow for greater system design simplicity. Previous demonstrations of wave sinks required the careful introduction of loss so that the scattering zero was brought to the real frequency axis where real frequency signals were then used, an example of CPA. By instead considering the use of complex frequency excitations, the ability to achieve sink‐like behavior could be unlocked in systems where the introduction of loss is not desirable.

Our work shows how any structure where an incoming monopolar signal is coupled solely to outgoing monopolar radiation (such as in any isotropic axisymmetric scatterer) can function as a complex frequency sink. This gives the techniques we have described straightforward applicability to a wide range of wave platforms. In particular, the ability to create a sink‐like effect through the use of a complex frequency input signal may unlock new capabilities in sensing and imaging applications. These results may also be fruitfully extended to asymmetric or non‐isotropic resonators where interesting directional or multipolar scattering effects could potentially be enabled by complex frequency signals.^[^ ^42^ ^]^

From an experimental standpoint, we have demonstrated a process for finding scattering singularities. Such methods are key to quickly and accurately characterize scattering systems, especially in the absence of accurate models or when numerical predictions are computationally expensive. We also note that, as the input energy is continuously funneled into the sink, very large wave amplitudes can be attained quickly and efficiently. This technique may then be profitably applied in situations where high field amplitudes are required. It is in this spirit that the effect of nonlinearities on the performance of such a sink could be fruitfully explored. Also, as discussed previously, the ability to create a subdiffraction focal point is of relevance for imaging applications. It has been shown, such as with Maxwell's lens, that a perfectly small spot size can be achieved, but only when the lens is accompanied by a wave sink,^[^ ^10^, ^20^, ^21^, ^43^ ^]^ which so far has required the tailored introduction of loss. Interestingly, our work hints that such a lens is possible even in the absence of loss by engaging specific complex frequencies.

## 4 Experimental Section

### Simulations

The finite element models used were 2D (1D axisymmetric) acoustic models in Comsol. The ambient medium was modeled as a circular domain of air of density 1.21 kg m^−3^ and speed of sound 343 m s^−1^. A circular inhomogeneity of radius 1 cm, density 74.6 kg m^−3^, and speed of sound 14.8 m/s was placed at the origin. The cylindrical wave radiation condition was used at the outer edge to launch incident waves of frequency 545.9 + 13.65*i* Hz while allowing outgoing radiation to leave the computational domain. The simulations were conducted in the time domain with the excitation signal specified as either a real or complex harmonic signal. The plotted results in Figure 3 are the pressure field maps of the entire domain at different instances in time.

### Experimental Setup

Experimental results were obtained using a square aluminum plate (Young's modulus 68.9 GPa, density 2700 kg m^−3^) with side length 36″ (0.9 m) and a thickness 0.19″ (4.8 mm). Circles of radius 0.5″ (12.7 mm) were milled out of the center of the plate on each side such that the thickness inside this circle is 0.05″ (1.27 mm). The plate was excited using a circle of 32 piezoelectric actuators (Steminc) attached 3.9″ (10 cm) away from the center of the plate using J‐B Weld ClearWeld epoxy. The smaller size of this circle relative to the entire plate helps prevent interference from waves reflecting off of the outer edge of the plate. Damping material was attached to the edges of the plate in order to further reduce external interference. The signal used to excite the plate is given by a cosine function multiplied by an exponential growth factor with a complex frequency of 17 + 1.4*i* kHz. This signal is also multiplied by a sigmoid function in the form of a hyperbolic tangent to minimize cut‐on and cut‐off effects. This signal was amplified with a Piezo Systems model EPA‐104 amplifier before being sent to the actuators. The response of the plate was recorded using a Polytec PSV‐500 laser Doppler vibrometer, with a sampling frequency of 390.625 kHz. The data was filtered using a band pass filter with cutoff frequencies at 10 and 30 kHz. Responses were recorded for 10,113 points distributed evenly across the inside of the circle of actuators. For each point, data was taken 40 times and averaged to reduce noise. This data was then combined to show the total response of the system.

### Separation of Incoming and Outgoing Signals

The recorded wavefield was separated into its incident and scattered components through Fourier transformation in polar coordinates. The domain of interest was defined within the circular region of radius *a* = 0.1 m, corresponding to where the piezoelectric patches were attached. A discrete Fourier transform (DFT) operation was performed within the MATLAB environment to transform the wavefield *w*(*r*, θ, *t*) into its reciprocal space representation $\hat{w}\left(k_{r},k_{\theta },\omega \right)$, where *k*_*r*_ and *k*_θ_ are the wavenumbers along the radial and angular directions, while ω denotes angular frequency. The reciprocal space representation of the wavefield was then separated into incident and scattered components; the quadrants defined by sign(*k*_*r*_ω) = −1 corresponded to radially outgoing waves, while sign(*k*_*r*_ω) = 1 to radially incoming waves, for all *k*_θ_. After such separation, the two components were converted back to their real space representation through an inverse DFT operation.

## Conflict of Interest

The authors declare no conflict of interest.

## Supporting information

Supporting Information

Supporting Information

Supporting Information

Supporting Information

Supporting Information

## Data Availability

The data that support the findings of this study are available from the corresponding author upon reasonable request.
