# Supplementary material for: A Lossless Sink Based on Complex Frequency Excitations
Source: Adv Sci (Weinh). 2023 Aug 16;10(28):2301811. doi: 10.1002/advs.202301811 (PMC10558693; doi:10.1002/advs.202301811)
Supplement: Supplementary file 1 — Supporting Information [file ADVS-10-2301811-s004.pdf]

ADVANCED SCIENCE
Open Access

Supporting Information

for *Adv. Sci.*, DOI 10.1002/advs.202301811

A Lossless Sink Based on Complex Frequency Excitations

*Curtis Rasmussen, Matheus I. N. Rosa, Jacob Lewton and Massimo Ruzzene**

Supplementary Information for: A loss and gain free sink based on complex frequency excitations

Curtis Rasmussen[1], Matheus I. N. Rosa[1], Jacob Lewton[1], and Massimo Ruzzene*[1]

[1]Department of Mechanical Engineering, University of Colorado Boulder, Boulder CO 80309

Supplementary Note 1: Description of Supplementary Movies 1 and 2

The animations show the total wave field when complex and real frequency waves are incident on an axisymmetric 2D resonator. The material properties for the resonator and surrounding medium are derived using the wavenumber and impedance ratio values from the main text. The finite element simulations are conducted using Comsol Multiphysics.

Supplementary Note 2: Description of Supplementary Movies 3 and 4

The videos show the measured wave field on the aluminum plate as recorded by a scanning laser Doppler vibrometer. Almost complete steady-state reflection seen in the case of the real frequency as the waves seem to lock in place, which is indicative of standing waves. For the complex frequency excitation, the waves are seen to almost uniformly enter the small resonator section until the signal is turned off at which point the waves are released.

Supplementary Note 3: 2D complex frequency sink theory

We consider the general case of a time-harmonic wave governed by the Helmholtz equation,

$$(\nabla^2 + k^2)\psi(\mathbf{r}, t) = 0, \tag{S1}$$

with ψ as the field variable at position $\mathbf{r}$ and time t. We consider the case of no dispersion, with the wavenumber given as $k = \omega/c$, where ω is the angular frequency and c is the wavespeed. Working in polar coordinates, we express the scattering from an inclusion in a homogeneous medium in terms of harmonic orders (monopole M, dipole D, and so on) with the scattering matrix S according to,

$$S \begin{pmatrix} M_{inc} \\ D_{inc} \\ \vdots \end{pmatrix} = \begin{pmatrix} M_{sc} \\ D_{sc} \\ \vdots \end{pmatrix}. \tag{S2}$$

To realize a sink, we deal with an eigensolution of this equation of the form

$$S \begin{pmatrix} M_{inc} \\ 0 \\ \vdots \end{pmatrix} = \sigma \begin{pmatrix} M_{inc} \\ 0 \\ \vdots \end{pmatrix}. \tag{S3}$$

In this formulation, the amount of monopolar scattering is the amount of monopolar radiation incident scaled by the eigenvalue: $M_{sc} = \sigma M_{inc}$. For there to be no scattering we need to find the condition that ensures $\sigma = 0$.

The field outside the resonator, ($r > a$, with r as the radial variable and a as the radius of the circular resonator), is a superposition of the incident and scattered monopolar waves, described by zero-order Hankel functions of the second and first kind, respectively,

$$\psi_0 = M_{inc} H_0^{(2)}(k_0 r) + \sigma M_{inc} H_0^{(1)}(k_0 r), \tag{S4}$$

using the $e^{-i\omega t}$ time convention. The subscript on ψ and k indicates the medium (0 for surrounding medium, 1 for the sink medium). Within the sink, we assume a standing wave solution described by a zero-order Bessel function with amplitude A_{sink},

$$\psi_1 = A_{sink} J_0(k_1 r). \tag{S5}$$

We enforce the boundary conditions,

$$\begin{aligned} \psi_0|_{r=a} &= \psi_1|_{r=a} \\ Z_1 k_1 \nabla \psi_0|_{r=a} &= Z_0 k_0 \nabla \psi_1|_{r=a}, \end{aligned} \tag{S6}$$

where Z_i is the free space impedance of medium i, and also impose the condition that the scattering vanishes, $\sigma = 0$. Taken together, these equations lead to

$$\frac{Z_0}{Z_1} = \frac{J_0(k_1 a) H_1^{(2)}(k_0 a)}{J_1(k_1 a) H_0^{(2)}(k_0 a)}. \tag{S7}$$

This is the relation between material parameters, sink size, and frequency of excitation that must be satisfied to realize the zero scattering effect. For lossless systems with real frequency excitation, all variables are real and no

solutions to the equation exist. However, solutions can be found by extending the wavenumbers into the complex plane which we do by considering complex frequency excitations.

To put the equation in terms of convenient dimensionless parameters we make the substitutions, $Z_r = Z_0/Z_1$, $\kappa_i = k'_i a$ (with $k' = \mathrm{Re}[k]$), and $R = \omega'/\omega''$:

$$Z_r = \frac{J_0(\kappa_1(1+i/R))H_1^{(2)}(\kappa_0(1+i/R))}{J_1(\kappa_1(1+i/R))H_0^{(2)}(\kappa_0(1+i/R))}, \quad \text{(S8)}$$

which is the equation arrived at in the main text. In this equation there are four variables. Figure S1 shows curves for different values of R that satisfy this equation. For the sink to be smaller than the wavelength of the oscillatory part of the complex frequency wave ($a/\lambda'_0 < 1$) we need $\kappa_0 < 2\pi$ since $\kappa_0 = k'_0 a = 2\pi a/\lambda'_0$. The red dot indicates the point in parameter space used in the main text. At this point $R = 40$, $\kappa_1 = 2.31$, $\kappa_0 = 0.1$, and $Z_r = 0.38$. The simulation results presented in the main text were from a 2D axisymmetric finite element model of acoustic waves. In this case the free space impedance is $Z_i = \rho_i c_i$, where ρ_i is the density of medium i and c_i the speed of sound of medium i. With the surrounding medium as air ($c_0 = 343$ m/s and $\rho_0 = 1.21$ kg/m^3) and the sink specified as having a radius $a = 1$ cm, the sink materials properties are then $c_1 = 14.83$ m/s and $\rho_1 = 74.59$ kg/m^3 so that there is no scattering for a complex frequency wave of the form $\mathrm{Re}[e^{i2\pi ft}]$ launched radially inward. The first complex frequency that achieves zero scattering is then $f = 545.9 + 13.65i$ Hz.

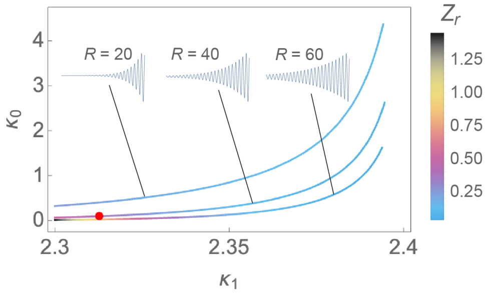

Figure S1: **Complex frequency sink parameter space.** Lines show solutions to Eq. (S8) with color indicating the impedance ratio required. Each line corresponds to a different ratio R between the real and imaginary parts of the required complex frequency excitation. The point in parameter space indicated by the red dot is what is used in the finite element simulations described in the main text.

Supplementary Note 4: Elastodynamic plate theory for a complex frequency sink

We demonstrate that the theory for the complex frequency sink can be applied to a variety of wave platforms by showing its applicability to waves in an elastic plate. Starting from the equations and solution ansatz described in the main text, the relevant boundary conditions at the resonator interface $r = a$ are the continuity of w, $\partial_r w$ (∂_r being the derivative with respect to the radial coordinate), bending moment $M_r = -D[\partial_r^2 w + (\nu/r)\partial_r w]$, and generalized Kirchhoff stress $V_r = -D\partial_r[(1/r)\partial_r(r\partial_r w)]$. With these boundary conditions, we have a set of four equations with the five wave amplitudes. For the complex frequency sink we are interested in the amount of scattering relative to the incident energy so we set $M_{sc} = \sigma M_{inc}$. We can now solve for σ in terms of the complex frequency ω contained in the wavenumbers $k_i(\omega)$. Figure S2 is a plot of σ in the complex frequency plane. With it we can find the discrete complex frequencies that result in zero scattering, clearly seen at three locations above the real frequency axis. As before, $|\sigma| = 1$ along the real axis as there is no dissipation, meaning that, for all real frequencies, all incident energy is scattered in the steady-state condition. The first zero is located at 19.7 + 2.4i kHz. This complex frequency, obtained under the assumptions of Kirchhoff theory, is used as a starting point in our search for the zero in our experimental setup. More accurate results were subsequently obtained from the real-frequency sweep detailed in Supplementary Note 5.

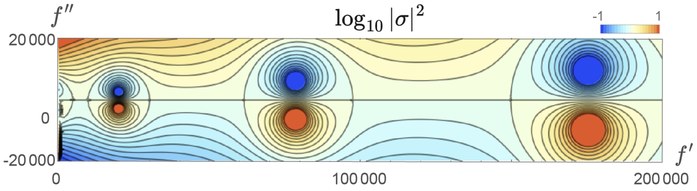

Figure S2: **Complex frequency landscape for elastic plate.** The first three scattering zeros are present in the upper half plane, corresponding to exponentially growing oscillatory signals. Such analysis is used to find feasible parameters such as plate dimensions and excitation signals that enable an experimental demonstration of a complex frequency sink for elastic waves.

In the broad context of guided waves in plates [1], the presented formulation based on Kirchoff theory is accurate for wavelengths that are large compared to the plate thickness, giving a good approximation of the A_0 (first asymmetric) lamb wave-mode. An extensional (S_0) mode is also present in the low-frequency regime, but is dominated by in-plane displacement components that are not excited or measured in our experimental setup. At higher frequencies, more accurate approximations of the (A_0) mode can be obtained using thick plate (Mindlin) theory [2]. Higher order bending modes (such as the A_1 mode) must

also be considered if operating at frequencies higher than the cut-on frequencies of these modes. In our experiment with an aluminum plate, the cut-on frequency of the A_1 mode is approximately 320 kHz, which means that considering only the A_0 mode is sufficient.

Supplementary Note 5: Experimental measurement to identify complex frequency zero

While simplified plate theory was used to find the general location of the complex frequency that would minimize scattering, a more accurate estimate of the complex frequency was obtained by probing the plate with real frequency excitations. As discussed in the main text, while the magnitude of the reflected signal is always one at real frequencies, the phase can take on any value and, in particular, there are phase swings at the locations of the critical complex frequencies. The locations of the phase swings indicates the real part of the critical complex frequencies and the widths of the phase swings indicates the imaginary part of these complex frequencies. Using our experimental setup, we send in a swept sine real frequency signal and monitor the reflected signal. We plot the phase of this reflected signal (solid line in Fig. S3) and observe the phase swing near 17 kHz. By comparing this response to the phase of a simple zero given by $(\omega - \omega_c)/(\omega - \omega_c^*)$, we find that a good fit is achieved for $f_c = \omega_c/2\pi = 16.8 + 0.7i$ kHz (dashed line in Fig. S3). We used this frequency as a starting point for our final experiment, where we manually searched the complex frequency space around this complex frequency until finding the complex frequency that minimized scattering at $17 + 1.4i$ kHz as discussed in the main text.

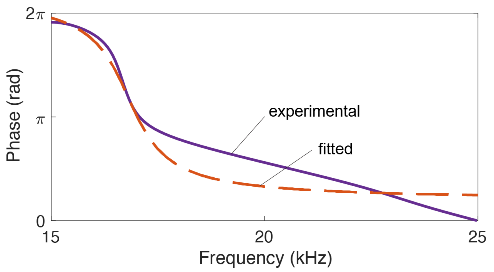

Figure S3: **Real frequency phase swing.** Phase of the reflected signal from a real frequency sweep excitation. An estimate of the location of the zero-reflection complex frequency location is obtained by fitting the measured curve to that of a simple resonance.

References

1. Graff, K. F. *Wave Motion in Elastic Solids* (Dover Publications, 1991).
2. Vemula, C. & Norris, A. N. Flexural wave propagation and scattering on thin plates using Mindlin theory. *Wave Motion* **26.** ISSN: 01652125 (1997).
