# Supplementary figures and images for: A Lossless Sink Based on Complex Frequency Excitations
Source: Adv Sci (Weinh). 2023 Aug 16;10(28):2301811. doi: 10.1002/advs.202301811 (PMC10558693; doi:10.1002/advs.202301811)

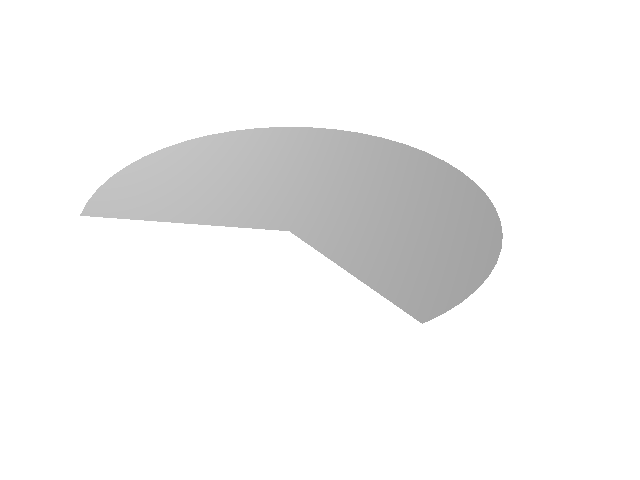

Supplement: Supplementary file 2 — Supporting Information [file ADVS-10-2301811-s005.gif]

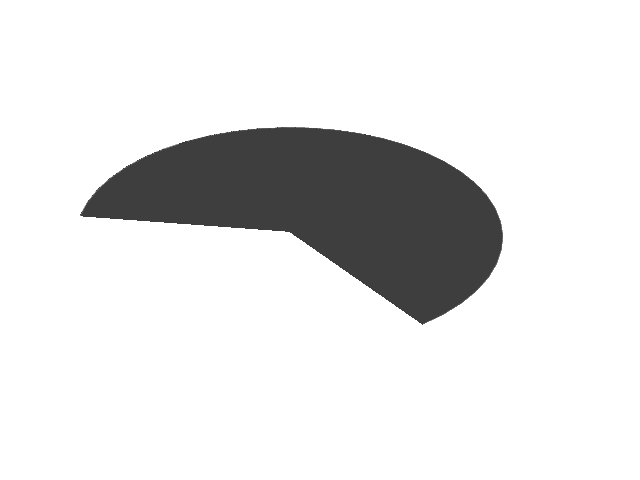

Supplement: Supplementary file 3 — Supporting Information [file ADVS-10-2301811-s002.gif]
